# The impact of online toxicity on the school ecosystem: an integrative review and conceptual framework

**DOI:** 10.3389/fpubh.2026.1783128

**Published:** 2026-08-17

**Authors:** Beth D. Marshall, Lauren M. Klein, Marina Jenkins, Sara B. Johnson

**Affiliations:** 1 Department of Population, Family and Reproductive Health, Johns Hopkins Bloomberg School of Public Health, Baltimore, MD, United States; 2 Department of Pediatrics, Johns Hopkins School of Medicine, Baltimore, MD, United States

**Keywords:** adolescents, cyberbullying, digital health, mental health, online toxicity, school climate, schools, teachers

## Abstract

The effects of online toxicity on children and adolescents permeate nearly every part of their lives. The harmful effects of online behavior and interactions increasingly impact not just students, but also teachers, school staff, and families, local education leaders, and even public confidence in education. Online toxicity is linked with serious mental health symptoms, school withdrawal and absenteeism, fighting and violence among students, and harassment of teachers, staff, and school board members. Increasingly, schools have looked to structural and policy interventions, including cell phone policies, to reduce the effects of negative online interactions on student behavior and learning during the school day; however, these approaches address only part of the problem. In this conceptual analysis, we begin by summarizing what is known about the impact of online toxicity on schools as organizations and the individuals who interact with them. We propose a conceptual framework illustrating how online behavior affects schools (e.g., peer–to–peer, student-to-teacher, family-to-staff, community-to-staff) to help explain this phenomenon. Finally, we discuss the strengths and limitations of current interventions and highlight opportunities to enhance interventions to support the health and well-being of students and school communities navigating online toxicity.

## Introduction

Online toxicity (also called digital toxicity, internet toxicity) encompasses a range of behaviors online that have direct and indirect negative consequences for students and schools. Online toxicity includes cyber-based threats, insults, harassment, obscenity, purposeful embarrassment, identity-based hate, stalking, and gender-based violence (1, 2). The most well-documented form of online toxicity is cyberbullying, which is increasingly prevalent and persistent in the lives of school-aged children. In a national sample of 13–17-year-olds in the United States (US) in 2023, 55% reported ever having been cyberbullied, more than double the rate in 2016 (27%) (3). In 2023, 27% of students reported experiencing cyberbullying in the past 30 days (3). Victims most often reported experiencing mean or hurtful comments, exclusion from group chats, rumors, or embarrassment/humiliation online (3). Adolescent girls were more likely to experience cyberbullying than boys (3).

At the student level, the impact of online toxicity manifests in a variety of ways, including fighting, harassment, exclusion, distraction, lack of engagement in the classroom, and school absence. Moreover, schools are systems; individuals inside the school interact amongst themselves (4) and, in turn, they interact with multiple systems outside the school (e.g., parents, policy makers, school board members, community members) (5). To date, most studies of the impact of online toxicity have focused on students, and a systems perspective is lacking. This narrow focus on students underestimates the potential full impact of online toxicity on schools; there are a variety of ways that online toxicity at one level can spill over to other parts of the system, with downstream effects on students’ experience in schools. For example, parents and family members may be drawn into physical conflicts stemming from students’ online interactions. Students and parents may harass, threaten, or intimidate teachers and school staff based on perceived unfair treatment or policies they oppose, and this may spill over into physical altercations (6, 7). Online harassment of teachers and resulting burnout and turnover could undermine classroom teaching and student/teacher relationships. Members of the broader community may harass or intimidate school leaders and school board members online to influence the operation of schools, districts (8, 9), the composition of school boards, and the educational system more broadly (8). As such, the impact of online toxicity can go well beyond negative student interactions.

Ecological Systems Theory is commonly used in child development and public health to describe how individual outcomes are influenced by multiple, nested, and interacting levels of influence, from individual to societal (10). This framework focuses attention on interactions between individuals and their environments, and can inform a more comprehensive approach to prevention and intervention across these levels. To date, no ecological conceptual frameworks for the impact of online toxicity on schools have been proposed to understand, organize, and guide new research and policy. In this paper, we aim to fill this gap. Guided by Ecological Systems Theory, we identify and synthesize U.S. peer-reviewed studies and gray literature on: (1) the impact of online toxicity on school communities, and (2) interventions to reduce or prevent its negative effects (10). After summarizing existing evidence, we recommended next steps for supporting the health and well-being of school communities informed by an ecological perspective.

## Developing and refining a conceptual framework

**Figure 1:**
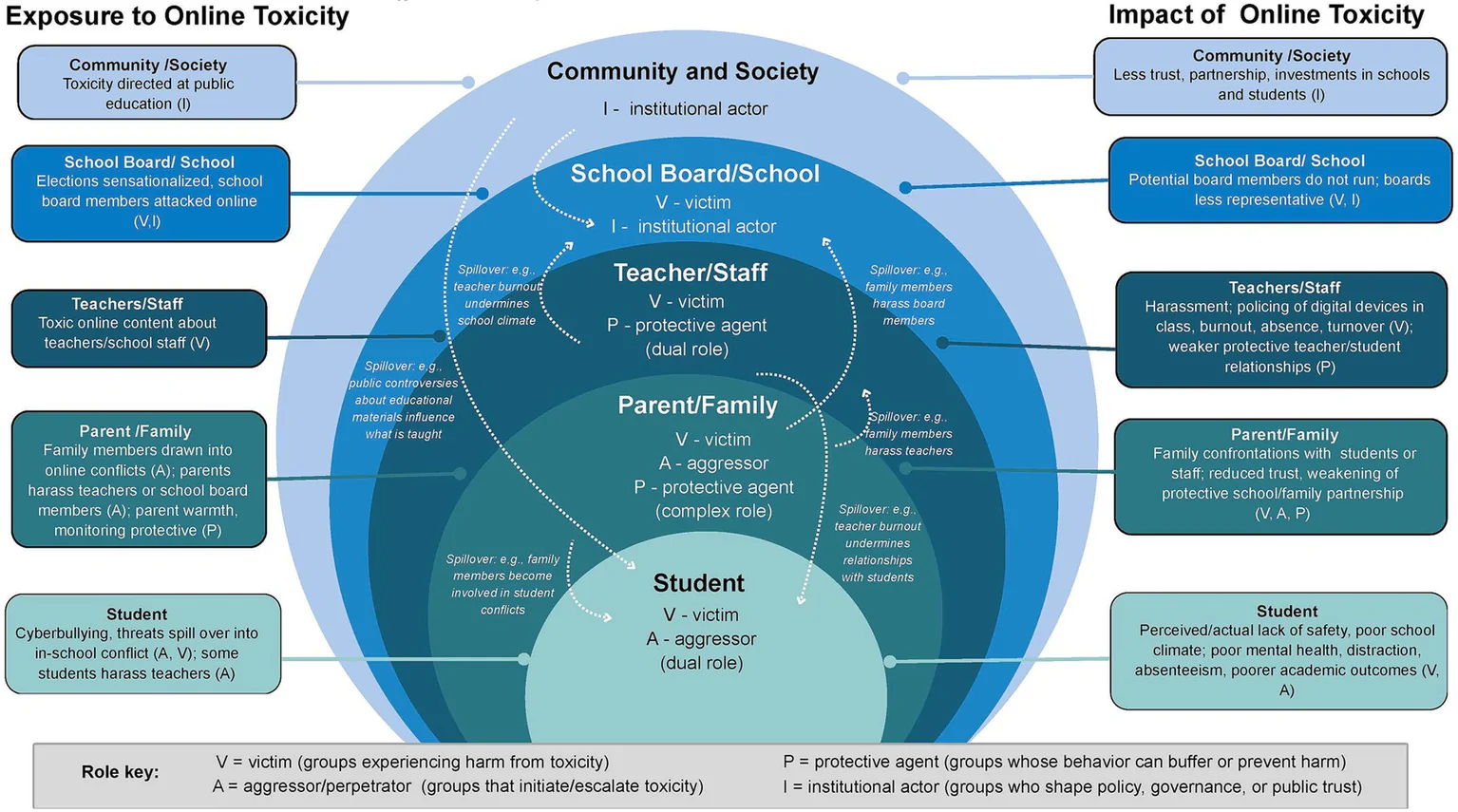
A conceptual model of the potential exposure and impact of online toxicity in the school ecosystem.

We began by constructing a guiding conceptual framework. We reviewed existing ecological frameworks that could inform the impact of online toxicity on schools (11–13) and used these frameworks to determine the levels of social ecology to be represented in our framework. Although frameworks characterized levels differently, they generally emphasized the need for consideration of individual, family and peer, school environment and policy, and broader social and policy factors (11–13). The model we created most closely mirrors the McLeroy et al. (11) Ecological Model for Health Promotion, which is comprised of the following levels: intrapersonal factors (i.e., individual characteristics), interpersonal processes and primary groups (e.g., families, peers), institutional factors (e.g., organizational characteristics), community factors (i.e., multiple institutions and organizations within a community), and public policy (at local, state, and national levels). We adapted this broad model specifically for the school context, drawing on the Coordinated School Health Program Ecological Model (12), which elucidates various specific components of school climate that we chose to capture via school and community/societal levels in our framework, and the Social-ecological Model of Cyberbullying (13), which emphasizes the role of norms, attitudes, and perceptions at different levels within the framework. In our final conceptual model (Figure 1), students are nested within parents/families, teachers/staff, schools, and communities/societies; the potential impact of online toxicity is evident at all levels. We then organized findings from our literature review into these nested levels of influence and refined the levels and their interactions based on existing evidence. When evidence from the peer-reviewed literature was sparse, we turned to the gray literature, which allowed us to identify potential impacts of online toxicity on schools that should be included in future research.

### Integrative review

**Figure 2:**
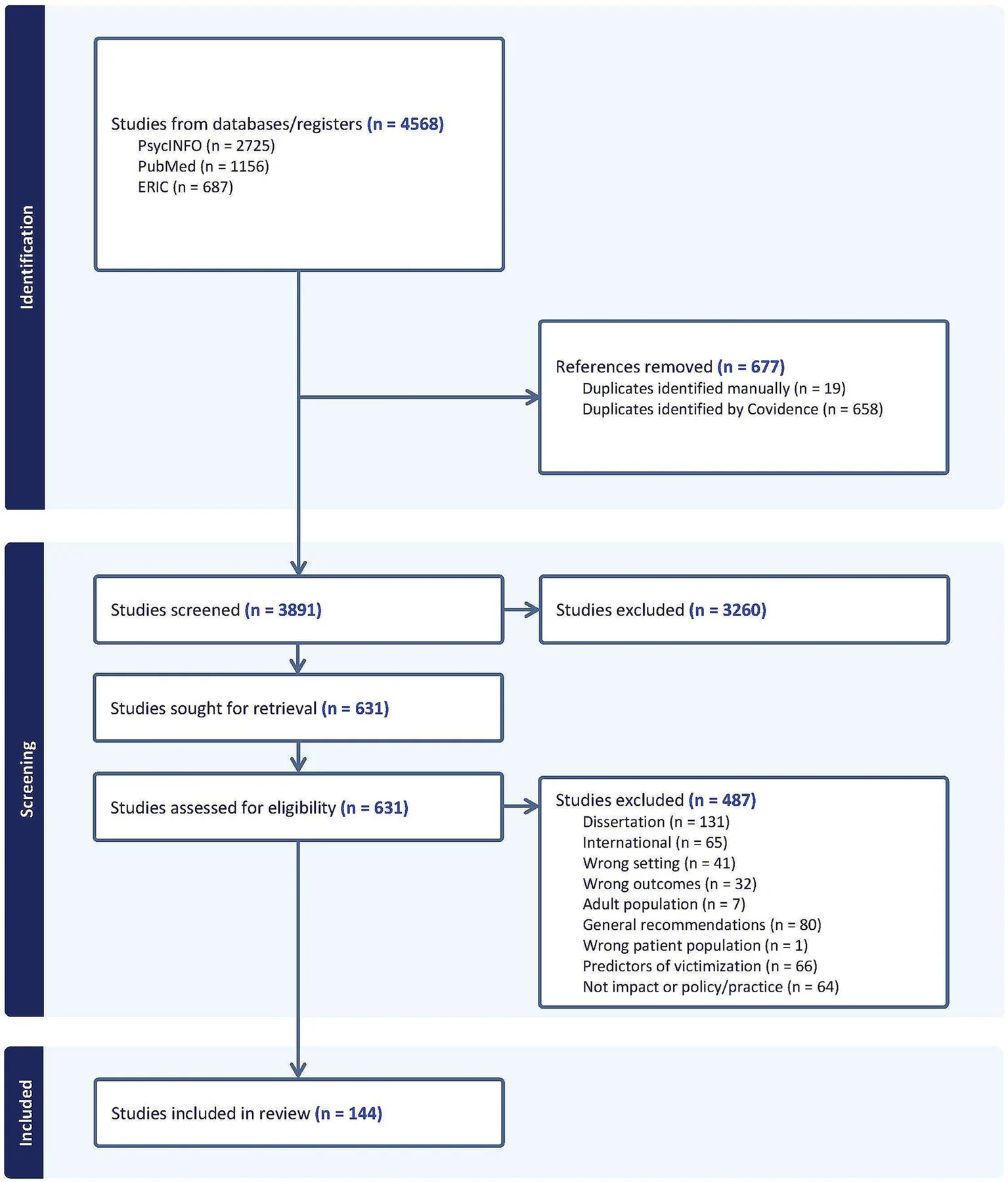
Online toxicity in schools search results.

Given the dearth of published research focused beyond the student level, we conducted an integrative review to identify evidence to inform a more robust understanding of the impact of online toxicity in schools (e.g., teachers/staff as victims, parents as aggressors, harassment of potential school board members). Integrative reviews can accommodate diverse methodologies and literatures to capture “context, processes, and subjective elements of the topic.” (14) First, we conducted a rapid review of the published literature using PubMed, ERIC, and PSYCINFO. We focused on manuscripts published between 2000 and 2025, in English, in the US, and focused on the impact of online toxicity on school communities or interventions to reduce or prevent these effects. Studies that focused on predictors of victimization or aggression online, “how to” or best practices tips for practitioners, and theses/dissertations were excluded (see Supplemental Table S1 for search strings). Single-reviewer voting was employed at the title and abstract and full-text review stages. At full-text review, studies were tagged at the ecological level by population. Of the 4,568 articles identified, 631 full texts were reviewed, and 144 were eligible for inclusion (see Figure 2 for details on search results and Supplemental Table S2 for the list of included articles). Data extraction was conducted using a standardized form in Excel. Reviewers extracted study design, outcomes, intervention or policy, population level, and any additional tags applied during full-text review for all included studies. Any disagreements during extraction were handled by consensus. We conducted supplemental searches using alternate search terms identified in our first search to ensure no impactful publications had been overlooked (e.g., reports, other gray literature, relevant literature outside of the US). Primarily informed by published literature and augmented gray literature, we constructed a conceptual framework to describe the impact of online toxicity on schools and opportunities for intervention.

**Figure 3:**
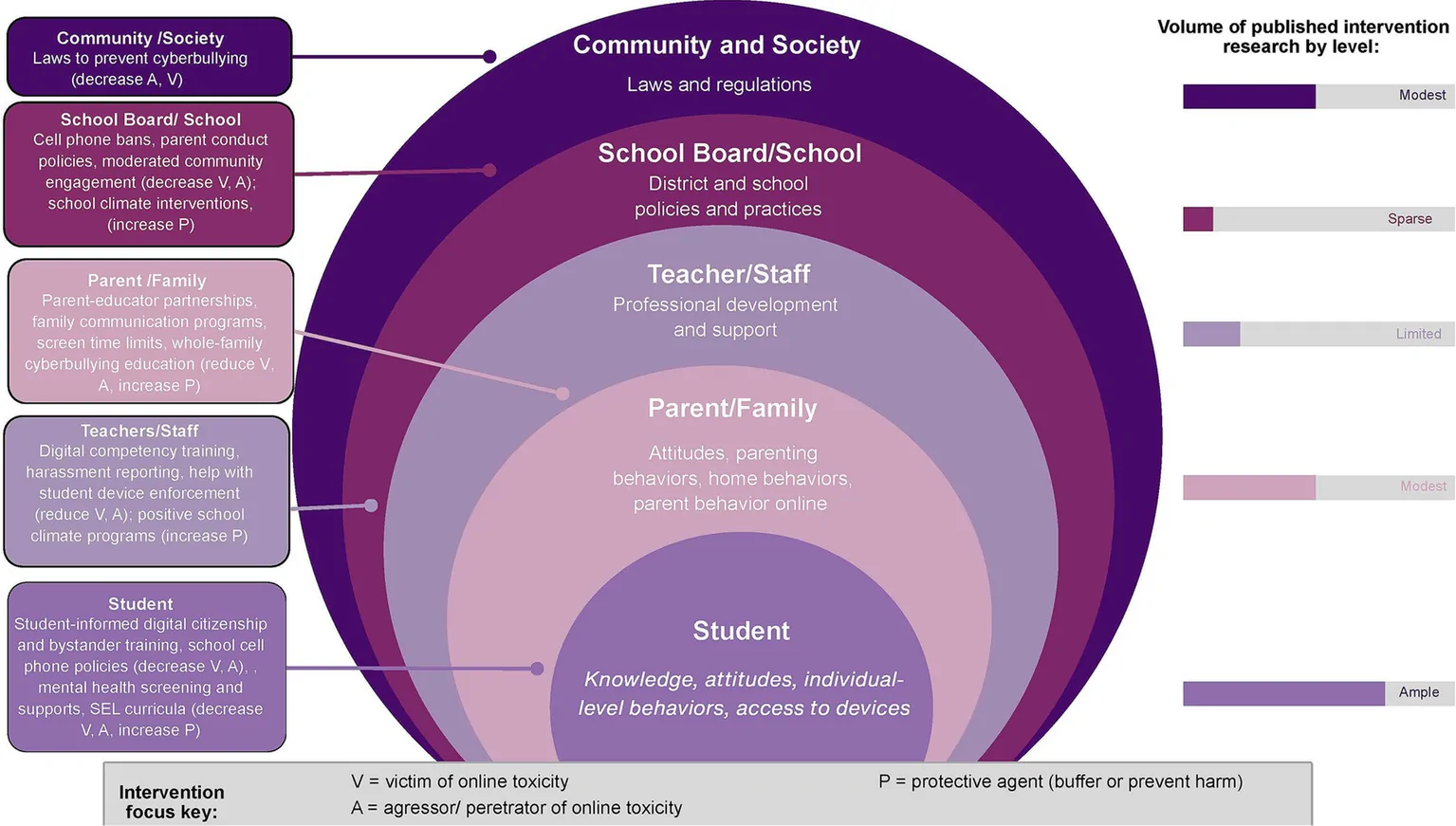
Multi-level intervention strategies for online toxicity.

Consistent with our conceptual framework, here, we summarize the results of the integrative review by levels of social ecology, informed as described above by previous research. First, we summarize evidence for the impact of online toxicity in each of these levels (Figure 1); this impact may differ across levels, ranging from individual health outcomes to organizational functioning to public trust, depending on the level of the framework being described. Additionally, although we present the evidence by level, we acknowledge that the levels represent the school ecosystem and are not separated from one another; as such, their impacts may cross over and interact. We then summarize the evidence for interventions to address the impact of online toxicity across levels (Figure 3).

## Introduction to the conceptual framework

Figure 1 presents an ecological framework that depicts the exposure to and impact of online toxicity across five nested levels of the school social ecology: Student, Parent/Family, Teacher/Staff, School Board/School, and Community and Society. For each level of the ecology, we indicate what roles that group commonly plays, including victim (V), aggressor (A), protective or buffering agent (P), and/or institutional actor (I); many groups occupy more than one role simultaneously. Boxes on the left side of the figure illustrate the forms of online toxicity exposure experienced at each level. Boxes on the right side of the figure illustrate the resulting impacts. Examples of potential spillover between levels are illustrated by curved arrows (e.g., family members being drawn into student conflicts, parents harassing teachers, and family members harassing school board members). The figure shows that online toxicity can operate across levels of the school ecosystem, with harms at one level creating downstream or upstream consequences at others.

## The impact of online toxicity across the social ecology of schools

### The impact of online toxicity on students

The impact of online toxicity on students is the level of the framework with the most peer-reviewed evidence. Most existing research focuses on cyberbullying (also referred to as cyber-victimization). Notably, cyberbullying may occur concurrently with in-school bullying (15–17), and their outcomes may be linked. As such, studies explore the impacts of each separately and/or together. In addition to cyberbullying, some studies examine online toxicity related to adolescent relationships, including cyber sexual harassment/violence (18, 19), cyber stalking (20), and cyber dating abuse victimization (21), as well as the effects of multiple types of online risk factors (cyberbullying, violence, drug-related, hate speech, profanity, sexual content, depression, and low-severity self-harm) (22).

Overall, evidence is generally limited to cross-sectional samples, thus limiting the ability to make inferences about the directionality of associations or mechanisms by which online toxicity contributes to various outcomes. Many studies are also limited by smaller or specific sample sizes, limiting generalizability of the results. Despite these notable limitations, research generally suggests a role of online toxicity in shaping student outcomes across multiple domains, including suicidality, internalizing symptoms (e.g., depression, anxiety), substance use, interpersonal violence, and academic outcomes.

There is overall support for an association between online toxicity and suicidality. A systematic review and meta-analysis drawing on findings from 25 articles on youth under age 25 found that cyberbullying victims and perpetrators had greater odds of suicidal behaviors and ideation (23). Nearly all studies were cross-sectional, with many using the national Youth Risk Behavior Survey (YRBS) to probe associations between cyberbullying/peer victimization and suicidal ideation and attempts. However, two notable exceptions, one longitudinal study (24) and one case–control study (22), suggest a temporal relationship wherein cyber risk preceded suicidal ideation. Several protective factors have been suggested to buffer the association between cyberbullying and suicidal and self-harm behaviors, including school connectedness, parenting practices and support, life satisfaction, and healthy diet (25).

Evidence from both cross-sectional and longitudinal studies supports a link between cybervictimization and internalizing symptoms (26–28), an association that might be compounded by in-person victimization (29). Other systematic reviews and longitudinal studies have found a positive association between cyberbullying, self-harm, and non-suicidal self-injury (30), particularly among those with more frequent bullying (31). Of note, longitudinal studies often have short follow-up periods (i.e., 6 months, 1 year) and small sample sizes, and some studies have found no association (32–34).

Longitudinal studies suggest online toxicity [cyberbullying (33, 35) and cyber dating abuse victimization (21)] is associated with increased substance use. One longitudinal study of cyber dating abuse victimization and hard drug and marijuana use noted that cross-sectional and longitudinal relationships between cyberbullying and substance use differed, suggesting the importance of exploring longitudinal effects to more accurately assess causal relationships (there were fewer significant associations in longitudinal analyses) (21). Other cross-sectional studies echo these results, finding an association between cybervictimization [cyber sexual harassment (18, 36), cyberbullying] and substance use. There is some evidence that greater perceived social support reduces the association between cyberbullying and substance use (37).

Studies have found associations of online toxicity with various outcomes related to interpersonal violence, including fighting (38) and weapon carrying (39). Other cross-sectional studies explored similar outcomes related to violence against others, with a few focusing on violent/aggressive behaviors more broadly (40, 41).

Research supports an association between online toxicity and various academic outcomes. A meta-analysis including 12 studies of adolescents found associations between cyber-victimization and attendance issues (*r* = 0.20) and poorer academic achievement (*r* = 0.14) (42). Although existing evidence is primarily cross-sectional, longitudinal studies have found evidence of a negative association between cyberbullying and student grades (38, 43), attendance (43), school dissatisfaction (44), school hassles (44), and perceived discrimination (44). One longitudinal study found an association between cyberstalking and academic performance, an association moderated by perceived social support from parents (20). Online toxicity was associated with feeling unsafe or fearful at school (45), sometimes to the point of missing school entirely (46), in cross-sectional studies, suggesting a potential mechanism by which online toxicity may be linked to academic consequences.

### The impact of online toxicity on parents and families

Most literature on the role of parents and families on school-related online toxicity focuses on the protective effects of positive parent–child relationships on risk of cyberbullying victimization, perpetration, and the negative health and well-being implications of cyberbullying experiences among students. Evidence shows that parental warmth, communication, and monitoring of adolescent online behaviors can all be protective in student experiences with cyberbullying (47, 48). Conversely, lack of parent–child communication around online behaviors and harassment, lack of parent involvement in adolescents’ online activity, and family conflict can increase the risks of adolescent cyberbullying victimization, perpetration, and related harms (48).

Few studies have examined parent involvement in school-related cyberbullying incidents among their children apart from household conditions and parental support. However, some studies have shown that parents report difficulties in working with schools to intervene in cyberbullying incidents, leading to distrust towards school staff and teachers (49). As there is some evidence that parent-educator collaboration is beneficial in cyberbullying prevention and intervention, it is important to develop and test strategies to align parent and school approaches and goals related to cyberbullying (50, 51). Similarly, some evidence suggests that adolescents do not want parents to be involved in school-related cyberbullying responses (52). One study found that although students and parents shared preferred approaches to cyberbullying, namely, ignoring the perpetrator, students expressed that they would rather approach a peer or teacher for advice rather than a parent (53). Other work describes parent-reported approaches to cyberbullying prevention and intervention, including familial communication, media use monitoring, and use of professional resources (54).

Studies also substantiate that parental online aggression towards teachers is prevalent, especially after the rise of online education following the COVID-19 pandemic (7, 55–57). Parent harassment of teachers and school administrators may be in response to student grades, teaching strategies and materials, or school policies. However, there is a lack of research around the impact of parent-involved online toxicity on students. There are documented cases of parent-involved perpetration of adolescent cyberbullying, illustrating a need for whole-family interventions to prevent and educate about cyberbullying and potential benefits of parent-educator partnership in these efforts (50).

### The impact of online toxicity on teachers and staff

Online toxicity directed at teachers and school staff in K-12 settings is a growing area of concern, but the peer-reviewed literature remains limited. In the US, national estimates for the prevalence of teacher online harassment are 6%, similar to reports of physical aggression toward teachers, and have increased over the last decade (7). Online aggression is often categorized with other types of verbal harassment, and researchers point to underreporting of online incidents, suggesting prevalence is underestimated (7). Data from the American Psychological Association Task Force’s violence against Educators and School Personnel survey reports online harassment with all reports of verbal or threatening victimization (56). Teachers face harassment from multiple sources, including students (59%), parents (63%), and, to a lesser degree, colleagues (49%). Parents are the primary perpetrators of verbal aggression toward school administrators (77%), followed by students (64%). These incidents increased post-pandemic for teachers and administrators; teachers experienced the largest increase (7). Data from case studies and open-ended national survey responses highlight growing online harassment from multiple sources. “A parent sent several threatening e-mails and Facebook® messages to me. She accused me of terrible things and told me that all the other students were not returning to our school unless I quit my job.” [The teacher] also described another incident, where “a former teacher is posting unprofessional things about [her] online and telling staff that she and another teacher were trying to get [her] fired.” (56, 58) Online harassment of teachers is significantly associated with teachers leaving the profession (7).

### Impact of online toxicity on school districts and school boards

The peer-reviewed literature on the impacts of online toxicity on school boards remains sparse. There are numerous accounts in the gray literature that provide anecdotal evidence of its effects on school boards and board members, and the need for future work prompted FBI investigations and even Supreme Court cases (59). A mixed-methods report from the Bridging the Divide Institute reported 36% of school board members nationally reported experiencing hostility in the last year. Qualitative reports primarily highlight online harassment (60). Almost 70% of school board members surveyed expressed a reluctance to engage in political activities such as tackling controversial topics or running for re-election (60). The Threats and Harassment dataset has shown a steady increase in harassment of public officials since 2022, when tracking started (61).

### Impact of online toxicity on school communities/society

School communities exist within broader socio-political and historical contexts; therefore, it is important to consider how online toxicity can influence them. Empirical research in this area is also limited, and findings are drawn from the gray literature. Several national polls show that confidence in public schools in the US is at historic lows. For example, 73% of respondents were dissatisfied with public education, up from 57% in 2001 (62). Similarly, only 13% of respondents gave the nation’s public schools a grade of A or B, half as many as in 2004 (26%) (63). There is some support for the role of online toxicity in undermining support for investments in public education, but most evidence is anecdotal. There is growing evidence that organized networks of individuals online foment dissent and outrage about politically charged issues in schools, such as the teaching of climate change, race, religion, sexuality, and gender identity, funded by “dark money networks.” (64) The goal is to destabilize public education by reducing trust and support.

## Interventions to prevent and address online toxicity in schools

Figure 3 is presented as a companion to Figure 1 and shows multi-level intervention approaches to address aspects of online toxicity across the same social ecology. Example interventions relevant to each level are provided, annotated with the role each strategy is designed to address (i.e., reducing harm to victims, reducing aggressor behavior, or strengthening protective or buffering factors). Bars on the right side of the figure provide a visual representation of the relative volume of published intervention research available at each level, ranging from ample at the student level to sparse at the school board level. The figure highlights the full range of leverage points available across the school ecosystem to guide future research and intervention development.

### Interventions focused on students

Consistent with the literature on the effects of online toxicity in schools, most intervention literature focuses on students, specifically targeting aspects of cyberbullying. Cyberbullying interventions at this level are diverse, leveraging various techniques such as curricula, group or individual targeted responses, psychoeducation, school climate/policy (discussed below), skill building, training, and media (65). Some skills commonly targeted by interventions include response strategy development, empathy building, effective communication, and goal setting, social–emotional competence, self-regulation, or online safety skills (65, 66). These techniques are commonly employed in interventions that raise awareness of cyberbullying, improve the skills of cyber-bystanders, and build resiliency. Raising awareness of cyberbullying among both students and teachers is often advocated, but evidence remains limited (67, 68). Cyber-bystander intervention training aims to decrease bullying and support victims by empowering youth to be active bystanders upon witnessing bullying, although research suggests limited effectiveness (69, 70). Finally, resiliency programming has been cited as a way to buffer the mental health effects of bullying on victims (71). Resiliency programming includes building capacity for positive adolescent relationships (72), increasing self-compassion and goal setting, and improving coping strategies (71).

Overall, many prior studies find cyberbullying interventions to be at least modestly effective in preventing cyberbullying perpetration and victimization (65, 66, 70, 73) and improving mental health (e.g., anxiety, depression) (73). However, many programs are evaluated in terms of attitudes and intentions regarding cyberbullying, rather than the behaviors themselves, raising questions about true impact (74). Evaluations of such programs are done with varying degrees of rigor but have been found to have low scientific merit overall (74, 75). Studies have noted the importance of considering families, mental health professionals, teachers, digital experts, school nurses, researchers, and the broader school community in future interventions (67, 71, 76). They also underscore the need to determine whether program impacts are sustained over time, since diminishing longitudinal effects were observed in some cases/programs (76) after program implementation was deprioritized (75).

### Interventions focused on parents/families

Prior studies indicate that parent-educator partnerships may be an effective approach to preventing and stopping bullying. This may include regular communication, mutual support, and shared decision-making (49, 50). Specific recommendations include tracking bullying instances in school and at home, assuring parents know who to contact when bullying instances occur, responding to incidents with education, training, or counseling, and aligning stances around zero tolerance for bullying with appropriate disciplinary measures in school and at home. Research also suggests that a combination of contextual family factors, such as parent–child relationship and communication, and practical family factors, such as household technology rules and parental internet mediation, may be more beneficial in addressing and preventing cyberbullying perpetration and victimization (48). There is also some evidence to suggest that parent educational workshops around cyberbullying from schools may be an effective intervention (77). There is little guidance around interventions to address parent-educator online toxicity, but this may involve similar approaches, including improved communication between parents and educators, providing educational anti-cyberbullying materials to parents, and regular measurement of school climate, including parent perspectives (57).

### Interventions focused on teachers/staff and school boards

Peer-reviewed literature provides no rigorous evaluations of interventions to address the impact of online toxicity on teachers, school administrators, and school boards. However, these individuals and groups often play a role in interventions aimed at addressing the impact of online toxicity on students, as mentioned above. Although not evaluated, the Department of Justice began tracking harassment of education officials in 2021, resulting in 22 reports of threats against officials and 6 cases referred to local law enforcement (78).

### Interventions focused on community/society

All US states have passed some form of general anti-bullying legislation, and research suggests that these have resulted in modest decreases in in-school bullying events, particularly among younger students (79). As of 2022, 48 states had anti-bullying laws that included provisions for cyberbullying, the best studied student-level indicator of online toxicity (80). However, not all such policies include restrictions on students’ off-campus behavior, limiting their impact (80). In states with laws that included provisions for cyberbullying, one study found that high school students were less likely to report having been cyberbullied, but effect sizes were small (marginal reductions of 1%); overall, however, evaluations of anti-bullying legislation remain sparse. Moreover, such laws can be difficult to evaluate because they may be associated with increased reporting due to greater awareness or policy implementation (81). Beyond legislation, in response to public concern about the toll on young social media users, some large social media and internet search companies (Meta, Google, Microsoft) have created policies and toolkits to reduce cyberbullying (82).

## Discussion

Online toxicity in schools is a systemic issue that has evolved far beyond peer-to-peer cyberbullying, although this topic remains the focus of most published literature in this area. Digital toxicity (including cyberbullying, harassment, and identity-based hate) creates a spillover effect that threatens to destabilize every level of the school community, from individual students to broader societal attitudes. Although the peer-reviewed literature primarily focuses on students, the prevalence of online toxicity has increased across all levels of the school ecosystem (3, 83, 84). Students experience mental health and academic impacts as well as behavioral risks (23, 35, 40, 43). Educators and school board members are leaving their positions and avoiding controversial topics (60). And, at the broadest level, online toxicity may contribute to diminished trust in the public education system (63). Current interventions, which focus almost exclusively on student-level cyberbullying, are only modestly effective, and few if any address the entire system, a limitation to their effectiveness (65, 67).

This analysis and the conceptual framework proposed here, while not a validated causal model, move beyond the traditional, narrow focus on peer-to-peer cyberbullying to offer a comprehensive framework to generate hypotheses and organize much-needed future research, practice, and policy focused on the impact of online toxicity on teachers, parents, school boards, and society. Student-level data is abundant; however, extant studies are limited by a focus on older students, a lack of assessment of behavioral outcomes, and cross-sectional designs. Additionally, there is a significant gap in research regarding other school community members. At the parent level, research confirms that parent–child communication is a protective factor, but there is a lack of research on the potential spillover effects of parent-modeled toxicity on student behavior and well-being. Current understanding of the impacts of online toxicity for teachers, school administrators, school boards, and the broader community, as well as the interactive effects between these levels, relies heavily on anecdotal accounts and popular press rather than rigorous empirical studies.

Peer-reviewed literature on online toxicity directed at K-12 teachers and administrators in the US is limited compared to global research. Globally, researchers separate cyberbullying directed at teachers from other types of victimization (58) and further categorize types of online aggression toward teachers, including hostile emails, public slander, denigration on forums and social media, creating fake profiles, sharing humiliating photos or videos (often recorded without consent, particularly on platforms like YouTube), and sending threats or blackmail. Researchers outside of the US have used longitudinal designs to clarify, for example, that experiencing workplace cyberbullying is negatively correlated with teacher positive mental health (60, 61). Researchers outside of the US have also explored interventions to support K-12 teachers and administrators and identified school-wide positive climate interventions that explicitly include teachers, as well as clear and consistent policies regarding teacher-parent communication, as supportive practices (85, 86). Administrative support for teachers can be both a protective factor and a way to de-escalate incidents of online harassment (86). Offering community opportunities for engagement, like a moderated social media site for the school community, can encourage positive online communication (87). Lastly, individual digital competency skills may protect teachers from online harassment and can lessen the mental health impact of teacher cyberbullying (4, 88). Teacher training and mentoring programs on digital competency can minimize online incidents of harassment and their impact on teachers’ mental health.

Some of the strategies for reducing the impact of online toxicity on teachers employed outside of the US could also be extrapolated to approaches to reduce impact on school boards in the US. Such strategies could include communication policies and opportunities for moderated online community engagement (87, 88). Additionally, when online toxicity involves violent threats, legal investigations and action are necessary (59). A focus on understanding and addressing online toxicity toward those who make educational policy and guide classroom activities is an urgent priority for future research in the US.

## Limitations

As suggested above, there are some limitations to consider in this analysis and conceptual framework construction. The quality of the empirical literature is mixed, which is indicative of the state of the field. The published literature is dominated by cross-sectional analysis of student-level data. Beyond the student level, there are relatively few empirical articles. To supplement the empirical literature, we included gray literature to identify evidence of emerging research areas; however, we did not conduct a structured review, and the results are limited and selective. Additionally, the integrative review was limited to US contexts only; there is innovative work globally that focuses on the impact of online toxicity for teachers and other school administrators.

### Applying the framework in research and practice

Our framework conceptualizes schools as systems, with levels of the social ecology from student to community, with complex interactions within and between levels that may shift over time and human development. Efforts to understand and reduce both online toxicity and its harmful effects on population health should focus on leveraging this framework for research and practice. Future work needs to involve a fundamental shift from treating online toxicity as a series of isolated student incidents to addressing it as a systemic public health and educational crisis that affects every level of the school environment.

There are also several opportunities to use this framework to improve research. Most extant research is cross-sectional, leaving the temporality of the relationship between online behavior and negative health outcomes and their reciprocity over time unclear. Future studies should prioritize longitudinal designs, carefully measure potential confounding factors (e.g., baseline mental health symptoms), and examine variation among relevant subgroups (e.g., sex, age). Studies that explore the impact of online toxicity on teachers, school administrators, and school board members, including its role in teacher retention and school board governance, are needed. Intervention evaluations should examine the impacts on multiple levels of the school ecosystem and should focus on outcomes beyond attitudes and intentions, capturing directly observed or measured online behavior whenever possible. Further, future research should aim to capture complex interactions among levels of influence. For example, quantitative and qualitative studies are needed to investigate the crossover effects between levels, such as how parent-modeled toxicity (i.e., parents harassing teachers online) influences their children’s behavior and well-being. This may result in additional attention to family- versus student-focused interventions. Finally, methodological approaches that account for and integrate complexity, such as network analysis and structural equation models, will lend rigor.

In practice, school staff and other stakeholders can map potential multilevel influences on online toxicity in their school to ensure that the most relevant opportunities for prevention and intervention are addressed. Intervention development and evaluation need to move beyond student-focused curricula and engage the entire school community, including mental health professionals, digital experts, and families, to create a whole family, whole school, whole community response. Potential areas include digital competency training for staff as well as students, explicit parent engagement in shared decision making at the school level, moderated engagement platforms and structured administrative support for teachers, school administrators and school board members who are experiencing online toxicity. Lastly, future policy work needs to address off-campus online behavior and provide clearer protections for teachers and staff who are currently facing rising rates of online aggression. Further, effective legislative and policy strategies, and/or greater enforcement of existing strategies, may help to disincentivize harassment of school board members and promote more productive dialogue. Informed by the proposed conceptual framework, policy must evolve to address the organizational and workforce threats identified in the review, including clear and consistent policies regarding teacher-parent communication to prevent harassment and de-escalate conflicts, and expansion of anti-bullying legislation to include teachers, school administration, and school board members.
