# Supplementary material for: The impact of online toxicity on the school ecosystem: an integrative review and conceptual framework
Source: Front Public Health. 2026 Aug 17;14:1783128. doi: 10.3389/fpubh.2026.1783128 (PMC13527669; doi:10.3389/fpubh.2026.1783128)
Supplement: Supplementary file 1 [file Table_1.DOCX]

Supplemental Table 1. Search Strings by Database*

| Online Toxicity Terms | School Terms |
| --- | --- |
| **PubMed** | |
| ((online[tw] OR digital[tw] OR "social media"[tw] OR cyber*[tw] OR technology[tw] OR phone*[tw]) AND "hate"[Mesh]) OR "Cyberbullying"[Mesh] OR Cyberbully*[tw] OR "Cyber bully*"[tw] OR "online bully*"[tw] OR "on-line bully"[tiab:~1] OR "on-line bullying"[tiab:~1] OR cyberstalk*[tw] OR "cyber stalk*"[tw] OR "online stalk"[tiab:~1] OR "online stalking"[tiab:~1] OR "online impersonation"[tiab:~1] OR "online impersonating"[tiab:~1] OR "on-line stalk"[tiab:~1] OR "on-line stalking"[tiab:~1] OR "on-line impersonation"[tiab:~1] OR "on-line impersonating"[tiab:~1] OR "non-consensual intimate imagery"[tiab:~1] OR ((online[tw] OR digital[tw] OR "social media"[tw] OR cyber*[tw] OR technology[tw] OR phone*[tw]) AND ("hate* speech"[tw] OR "hateful comment*"[tw] OR "discriminatory content"[tiab:~1])) OR "online manipulation"[tw] OR trolling[tw] OR "toxic user behavior patterns"[tiab:~1] OR "cyberaggression"[tw] OR "cybervictimization"[tw] OR "cyber-aggression"[tw] OR "cyber-victimization"[tw] | "School Teachers"[Mesh] OR "Educational Personnel"[Mesh:NoExp] OR "Schools"[Mesh:NoExp] OR school*[tw] OR "school principal*"[tw] OR "school board*"[tw] OR "school administrat*"[tw] OR teacher*[tw] OR educator*[tw] OR instructor*[tw] OR staff[tw] |
| **ERIC** | |
| DE "Cyberbullying" OR ((DE "Hate Crime (2004)" OR "Hate") AND (online OR digital OR “social media” OR cyber OR technology OR phone)) OR "Cyberbullying" OR Cyberbully* OR "Cyber bully*" OR "online bully*" OR "on-line bully" OR "on-line bullying" OR cyberstalk* OR "cyber stalk*" OR "online stalk" OR "online stalking" OR "online impersonation" OR "online impersonating" OR "on-line stalk" OR "on-line stalking" OR "on-line impersonation" OR "on-line impersonating" OR "non-consensual intimate imagery" OR (("hate* speech" OR "hate* comment*" OR "discriminatory content") AND (online OR digital OR “social media” OR cyber OR technology OR phone)) OR "online manipulation" OR trolling OR "toxic user behavior pattern" OR "toxic user behavior patterns")) | ((DE "Teachers" OR (DE "Principals" OR DE "Assistant Principals" OR DE "Administrators" OR DE "Assistant Principals" OR DE "Superintendents" OR DE "School Boards" OR DE "Boards of Education" OR DE "State Boards of Education" OR DE "State School Boards" OR DE "State Departments of Education" OR DE "Board of Education Policy" OR DE "Governing Boards" OR DE "Board Candidates")) OR ((teacher* or educator* or instructor* or staff or faculty OR principal* OR administrator* OR "school board*"))) |
| **PsycINFO**** | |
| (DE "Cyberbullying" OR (DE "Hate" AND (online OR digital OR “social media” OR cyber OR technology OR phone)) OR (Cyberbullies OR "cyber bullies" OR Cyberbully* OR "Cyber bully*" OR "online bullies" OR "online bully*" OR "on-line bully*" OR cyberstalk* OR "cyber stalk*" OR (online N2 stalk*) OR (online N2 impersonat*) OR (on-line N2 stalk*) OR (on-line N2 impersonat*) OR doxing OR outing OR swatting OR extortion OR extorting OR "non-consensual intimate imagery" OR (("hate* speech" OR "hateful comment*" OR "discriminatory content") AND (online OR digital OR “social media” OR cyber OR technology OR phone)) OR "online manipulation" OR trolling OR "addictive digital behavior*" OR "compulsive digital behavior*" OR "toxic user behavior pattern*" OR "cyberaggression" OR "cybervictimization" OR "cyber-aggression" OR "cyber-victimization")) | ((DE "Charter Schools" OR DE "Elementary Schools" OR DE "High Schools" OR DE "Junior High Schools" OR DE "Middle Schools" OR DE "School Violence") OR (DE "Elementary School Teachers" OR DE "High School Teachers" OR DE "Junior High School Teachers" OR DE "Middle School Teachers" OR DE "Preservice Teachers" OR DE "Resource Teachers" OR DE "Special Education Teachers" OR DE "Student Teachers" OR DE "Vocational Education Teachers") OR (DE "School Administrators" OR DE "Boards of Education" OR DE "School Principals" OR DE "School Superintendents") OR (school* OR teacher* OR administrator* OR board OR boards OR principal* OR superintendent* OR educator* OR instructor* OR staff)) |

*All searches limited to English and used AND operator with Online Toxicity and School Terms.

** PsycINFO also limited by school age OR adolescence OR adulthood and 2000-2025.
