# Supplementary material for: The impact of online toxicity on the school ecosystem: an integrative review and conceptual framework
Source: Front Public Health. 2026 Aug 17;14:1783128. doi: 10.3389/fpubh.2026.1783128 (PMC13527669; doi:10.3389/fpubh.2026.1783128)
Supplement: Supplementary file 2 [file Table_2.DOCX]

**Supplemental Table 2. Studies From Rapid Review (N=144).**

| **Citation** | **Student** | **Parent/Family** | **Teacher/Staff** | **School Board** | **Community and Society** |
| --- | --- | --- | --- | --- | --- |
| Kurnaz MF, Koçtürk N. Addressing cyberbullying in adolescents: A comprehensive meta-analytic evaluation of intervention programs. Aggression and violent behavior. 2025 Aug 29:102081. | X |  |  |  |  |
| Zou W, Yang Q, DiFranzo D, Chen M, Hui W, Bazarova NN. Social Media Co-pilot: Designing a chatbot with teens and educators to combat cyberbullying. International Journal of Child-Computer Interaction. 2024 Sep 1;41:100680. | X |  |  |  |  |
| Lekamge RB, Jain R, Sheen J, Solanki P, Zhou Y, Romero L, Barry MM, Chen L, Karim MN, Ilic D. Systematic review and meta-analysis of the effectiveness of whole-school interventions promoting mental health and preventing risk behaviours in adolescence. Journal of youth and adolescence. 2025 Feb;54(2):271-89. | X |  | X |  |  |
| Jones LM, Mitchell KJ, Beseler CL. The impact of youth digital citizenship education: Insights from a cluster randomized controlled trial outcome evaluation of the be internet awesome (BIA) curriculum. Contemporary School Psychology. 2024 Dec;28(4):509-23. | X |  | X |  |  |
| Chicote-Beato M, González-Víllora S, Bodoque-Osma AR, Navarro R. Cyberbullying intervention and prevention programmes in Primary Education (6 to 12 years): A systematic review. Aggression and Violent Behavior. 2024 Jul 1;77:101938. | X |  | X |  |  |
| Ansary NS. Cyberbullying and best practices for prevention. InHandbook of Anger, Aggression, and Violence 2023 Jan 24 (pp. 1-25). Cham: Springer International Publishing. | X | X | X | X | X |
| Torgal C, Espelage DL, Polanin JR, Ingram KM, Robinson LE, El Sheikh AJ, Valido A. A meta-analysis of school-based cyberbullying prevention programs’ impact on cyber-bystander behavior. School psychology review. 2023 Mar 4;52(2):95-109. | X |  |  |  |  |
| Wright MF, Wachs S, Gámez-Guadix M. The role of perceived gay-straight alliance social support in the longitudinal association between homophobic cyberbullying and LGBTQIA adolescents’ depressive and anxiety symptoms. Journal of youth and adolescence. 2022 Jul;51(7):1388-96. | X |  |  |  |  |
| Polanin JR, Espelage DL, Grotpeter JK, Ingram K, Michaelson L, Spinney E, Valido A, Sheikh AE, Torgal C, Robinson L. A systematic review and meta-analysis of interventions to decrease cyberbullying perpetration and victimization. Prevention Science. 2022 Apr;23(3):439-54. | X |  |  |  |  |
| Vivolo-Kantor AM, Niolon PH, Estefan LF, Le VD, Tracy AJ, Latzman NE, Little TD, Lang KM, DeGue S, Tharp AT. Middle school effects of the Dating Matters® comprehensive teen dating violence prevention model on physical violence, bullying, and cyberbullying: A cluster-randomized controlled trial. Prevention science. 2021 Feb;22(2):151-61. | X |  |  |  |  |
| Sullivan TN, Farrell AD, Sutherland KS, Behrhorst KL, Garthe RC, Greene A. Evaluation of the Olweus Bullying Prevention Program in US urban middle schools using a multiple baseline experimental design. Prevention Science. 2021 Nov;22(8):1134-46. | X |  |  |  |  |
| Adkins V, Selkie E. Digital tools that promote or alleviate interpersonal violence. InTechnology and Adolescent Health 2020 Jan 1 (pp. 347-363). Academic Press. | X |  |  |  |  |
| Acosta J, Chinman M, Ebener P, Malone PS, Phillips A, Wilks A. Evaluation of a whole-school change intervention: Findings from a two-year cluster-randomized trial of the restorative practices intervention. Journal of youth and adolescence. 2019 May 1;48(5):876-90. | X |  | X |  |  |
| Salazar LR, Roberto AJ, Eden J, Savage M, Deiss D. A short intervention on cyberbullying for students in middle school and their parents. Reducing Cyberbullying in Schools. 2018 Jan 1:245-54. | X | X |  |  |  |
| Hatzenbuehler ML, Flores JE, Cavanaugh JE, Onwuachi-Willig A, Ramirez MR. Anti-bullying policies and disparities in bullying: A state-level analysis. American journal of preventive medicine. 2017 Aug 1;53(2):184-91. |  |  |  |  | X |
| Espelage DL, Low S, Van Ryzin MJ, Polanin JR. Clinical trial of second step middle school program: Impact on bullying, cyberbullying, homophobic teasing, and sexual harassment perpetration. School Psychology Review. 2015 Dec 1;44(4):464-79. | X |  |  |  |  |
| Della Cioppa V, O'Neil A, Craig W. Learning from traditional bullying interventions: A review of research on cyberbullying and best practice. Aggression and Violent Behavior. 2015 Jul 1;23:61-8. | X |  |  |  |  |
| Roberto AJ, Eden J, Savage MW, Ramos-Salazar L, Deiss DM. Outcome evaluation results of school-based cybersafety promotion and cyberbullying prevention intervention for middle school students. Health communication. 2014 Nov 26;29(10):1029-42. | X |  |  |  |  |
| Nocentini A, Zambuto V, Menesini E. Anti-bullying programs and Information and Communication Technologies (ICTs): A systematic review. Aggression and Violent Behavior. 2015 Jul 1;23:52-60. | X |  |  |  |  |
| Fraguas D, Díaz-Caneja CM, Ayora M, Durán-Cutilla M, Abregu-Crespo R, Ezquiaga-Bravo I, Martin-Babarro J, Arango C. Assessment of school anti-bullying interventions: A meta-analysis of randomized clinical trials. JAMA pediatrics. 2021 Jan;175(1):44-55. | X |  | X |  |  |
| Tozzo P, Cuman O, Moratto E, Caenazzo L. Family and educational strategies for cyberbullying prevention: A systematic review. International Journal of Environmental Research and Public Health. 2022 Aug 22;19(16):10452. | X | X | X |  |  |
| Henares-Montiel J, Pastor-Moreno G, Ramirez-Saiz A, Rodriguez-Gomez M, Ruiz-Perez I. Characteristics and effectiveness of interventions to reduce cyberbullying: a systematic review. Frontiers in public health. 2023 Aug 30;11:1219727. | X |  |  |  |  |
| Dailey SF, Roche RR. The SHIELD framework: Advancing strength-based resilience strategies to combat bullying and cyberbullying in youth. International Journal of Environmental Research and Public Health. 2025 Jan 7;22(1):66. | X | X | X |  | X |
| Lancaster M. A systematic research synthesis on cyberbullying interventions in the United States. Cyberpsychology, Behavior, and Social Networking. 2018 Oct 1;21(10):593-602. | X |  |  |  |  |
| Scheithauer H, Tsorbatzoudis H, editors. Guest editorial: Efficacious and effective preventive interventions to tackle cyberbullying in the school environment. Aggressive Behavior. 2016 Mar;42(2):111-3. | X |  |  |  |  |
| Cantone E, Piras AP, Vellante M, Preti A, Daníelsdóttir S, D’Aloja E, Lesinskiene S, Angermeyer MC, Carta MG, Bhugra D. Interventions on bullying and cyberbullying in schools: A systematic review. Clinical practice and epidemiology in mental health: CP & EMH. 2015 Feb 26;11(Suppl 1 M4):58. |  |  | X |  |  |
| Bickham DS, Moukalled S, Inyart HK, Zlokower R. Evaluating a middle-school digital citizenship curriculum (screenshots): Quasi-experimental study. JMIR Mental Health. 2021 Sep 15;8(9):e26197. | X |  |  |  |  |
| Ramirez M, Ten Eyck P, Peek-Asa C, Onwuachi-Willig A, Cavanaugh JE. Evaluation of Iowa’s anti-bullying law. Injury epidemiology. 2016 Jun 7;3(1):15. |  |  |  |  | X |
| Hamm MP, Newton AS, Chisholm A, Shulhan J, Milne A, Sundar P, Ennis H, Scott SD, Hartling L. Prevalence and effect of cyberbullying on children and young people: A scoping review of social media studies. JAMA pediatrics. 2015 Aug;169(8):770-7. | X |  |  |  |  |
| Baidoo CE, Alvarez MJ, Hawkins SS. The impact of cyberbullying laws on student cyberbullying by sexual minority status. Preventive Medicine Reports. 2025 Aug 25:103221. | X |  |  |  | X |
| Yosep I, Hikmat R, Mardhiyah A, Hazmi H, Hernawaty T. Method of nursing interventions to reduce the incidence of bullying and its impact on students in school: a scoping review. InHealthcare 2022 Sep 22 (Vol. 10, No. 10, p. 1835). MDPI. |  | X | X |  |  |
| Felk J, Liesegang J, Schmidt F, Donat M, Wolgast A. Verbal Patterns and Effective Strategies in Cyberbullying Prevention and Intervention: A Systematic Review. Child Psychiatry & Human Development. 2025 Sep 19:1-3. | X |  |  |  |  |
| Hatzenbuehler ML, Schwab-Reese L, Ranapurwala SI, Hertz MF, Ramirez MR. Associations between antibullying policies and bullying in 25 states. JAMA pediatrics. 2015 Oct;169(10):e152411. |  |  |  |  | X |
| Brown K, Jackson M, Cassidy W. Cyber-bullying: Developing policy to direct responses that are equitable and effective in addressing this special form of bullying. Canadian Journal of Educational Administration and Policy. 2006(57). |  |  | X | X | X |
| Eanes S. Cyberbullying and Cell Phone Policy in US Primary and Secondary Schools. Data Point. NCES 2019-053. National Center for Education Statistics. 2019 Jan. |  |  | X | X |  |
| Eldridge MA, Jenkins LN. The bystander intervention model: Teacher intervention in traditional and cyber bullying. International journal of bullying prevention. 2020 Dec;2(4):253-63. |  |  | X |  |  |
| Evangelio C, Rodríguez-González P, Fernández-Río J, Gonzalez-Villora S. Cyberbullying in elementary and middle school students: A systematic review. Computers & Education. 2022 Jan 1;176:104356. | X |  | X |  |  |
| Kim J, Walsh E, Pike K, Thompson EA. Cyberbullying and victimization and youth suicide risk: The buffering effects of school connectedness. The journal of school nursing. 2020 Aug;36(4):251-7. | X |  | X |  |  |
| Kasturiratna KS, Hartanto A, Chen CH, Tong EM, Majeed NM. Umbrella review of meta-analyses on the risk factors, protective factors, consequences and interventions of cyberbullying victimization. Nature Human Behaviour. 2025 Jan;9(1):101-32. | X |  |  |  |  |
| Aboujaoude E, Savage MW, Starcevic V, Salame WO. Cyberbullying: Review of an old problem gone viral. Journal of adolescent health. 2015 Jul 1;57(1):10-8. | X |  |  |  |  |
| Roberto AJ, Eden J, Deiss DM, Savage MW, Ramos-Salazar L. The short-term effects of a cyberbullying prevention intervention for parents of middle school students. International journal of environmental research and public health. 2017 Sep;14(9):1038. |  | X |  |  |  |
| Agley J, Jun M, Eldridge L, Agley DL, Xiao Y, Sussman S, Golzarri-Arroyo L, Dickinson SL, Jayawardene W, Gassman R. Effects of ACT Out! Social issue theater on social-emotional competence and bullying in youth and adolescents: Cluster randomized controlled trial. JMIR mental health. 2021 Jan 6;8(1):e25860. | X |  |  |  |  |
| Agatston P, Limber S. Cyberbullying prevention and intervention: Promising approaches and recommendations for further evaluation. InBullying prevention and intervention at school: Integrating theory and research into best practices 2018 Aug 29 (pp. 73-93). Cham: Springer International Publishing. | X |  |  |  |  |
| Agatston PW, Kowalski R, Limber S. Students’ perspectives on cyber bullying. Journal of Adolescent Health. 2007 Dec 1;41(6):S59-60. | X |  |  |  |  |
| Keith S, Scheuerman HL, Smith AL. Going viral: Investigating the short-and long-term traumatic effects of cyberbullying victimization on adolescents in schools. Psychological Trauma: Theory, Research, Practice, and Policy. 2025 Mar 31. | X |  |  |  |  |
| Thomas A, Van Puymbroeck M, Crowe BM, Townsend J, Farnsworth II JL. Identifying the relationship between recreation engagement, bullying, and suicidality in high school students. Children and Youth Services Review. 2024 Sep 1;164:107857. | X |  |  |  |  |
| Rostam-Abadi Y, Stefanovics EA, Potenza MN. An exploratory study of the prevalence and adverse associations of in-school traditional bullying and cyberbullying among adolescents in Connecticut. Journal of psychiatric research. 2024 May 1;173:372-80. | X |  |  |  |  |
| Lydecker JA, Winschel J, Gilbert K, Cotter EW. School absenteeism and impairment associated with weight bullying. Journal of adolescence. 2023 Oct;95(7):1478-87. | X |  |  |  |  |
| Guo S. Cyberbullying and delinquency in adolescence: the potential mediating effects of social attachment and delinquent peer association. Journal of interpersonal violence. 2022 Oct;37(19-20):NP18837-64. | X |  |  |  |  |
| Chacon M, Raj A. The association between bullying victimization and fighting in school among US high school students. Journal of interpersonal violence. 2022 Nov;37(21-22):NP20793-815. | X |  |  |  |  |
| Aguayo L, Beach LB, Wang X, Ruprecht MM, Felt D, Kershaw KN, Davis MM, Phillips G. Someone to talk to: the association of mentorship and cyberbullying with suicidality among US high school students. Social psychiatry and psychiatric epidemiology. 2022 Jan;57(1):149-59. | X |  | X |  |  |
| McCuddy T, Esbensen FA. Delinquency as a response to peer victimization: The implications of school and cyberbullying operationalizations. Crime & Delinquency. 2022 May;68(5):760-85. | X |  |  |  |  |
| Sutter CC, Stickl Haugen J, Campbell LO, Tinstman Jones JL. School and electronic bullying among adolescents: Direct and indirect relationships with sadness, sleep, and suicide ideation. Journal of Adolescence. 2023 Jan;95(1):82-96. | X |  |  |  |  |
| Kostyrka‐Allchorne K, Stoilova M, Bourgaize J, Rahali M, Livingstone S, Sonuga‐Barke E. Digital experiences and their impact on the lives of adolescents with pre‐existing anxiety, depression, eating and nonsuicidal self‐injury conditions–a systematic review. Child and Adolescent Mental Health. 2023 Feb;28(1):22-32. | X |  |  |  |  |
| Williford A, Fite P, Diaz K, Singh A. Associations between different forms of peer victimization and school absences: The moderating role of teacher attachment and perceived school safety. Psychology in the Schools. 2021 Jan;58(1):185-202. | X |  | X |  |  |
| Strohacker E, Wright LE, Watts SJ. Gender, bullying victimization, depressive symptoms, and suicidality. International journal of offender therapy and comparative criminology. 2021 Aug;65(10-11):1123-42. | X |  |  |  |  |
| Mitchell KJ, Jones LM, Turner HA. Past year technology-involved peer harassment victimization and recent depressive symptoms and suicide ideation among a national sample of youth. Journal of interpersonal violence. 2021 Feb;36(3-4):NP1165-1179NP. | X |  |  |  |  |
| Kwan I, Dickson K, Richardson M, MacDowall W, Burchett H, Stansfield C, Brunton G, Sutcliffe K, Thomas J. Cyberbullying and children and young people's mental health: a systematic map of systematic reviews. Cyberpsychology, Behavior, and Social Networking. 2020 Feb 1;23(2):72-82. | X |  |  |  |  |
| Yoon Y, Lee JO, Cho J, Bello MS, Khoddam R, Riggs NR, Leventhal AM. Association of cyberbullying involvement with subsequent substance use among adolescents. Journal of Adolescent Health. 2019 Nov 1;65(5):613-20. | X |  |  |  |  |
| Morin HK, Bradshaw CP, Kush JM. Adjustment outcomes of victims of cyberbullying: The role of personal and contextual factors. Journal of school psychology. 2018 Oct 1;70:74-88. | X |  |  |  |  |
| Lu Y, Avellaneda F, Torres ED, Rothman EF, Temple JR. Adolescent cyberbullying and weapon carrying: Cross-sectional and longitudinal associations. Cyberpsychology, Behavior, and Social Networking. 2019 Mar 1;22(3):173-9. | X |  |  |  |  |
| Wright MF. Adolescents’ cyber aggression perpetration and cyber victimization: The longitudinal associations with school functioning. Social Psychology of Education. 2015 Dec;18(4):653-66. | X |  |  |  |  |
| Williford A, Orsi R, DePaolis KJ, Isen D. Cyber and traditional peer victimization: Examining unique associations with children’s internalizing difficulties. Children and Youth Services Review. 2018 Oct 1;93:51-9. | X |  |  |  |  |
| Reed E, Salazar M, Behar AI, Agah N, Silverman JG, Minnis AM, Rusch ML, Raj A. Cyber sexual harassment: Prevalence and association with substance use, poor mental health, and STI history among sexually active adolescent girls. Journal of adolescence. 2019 Aug 1;75:53-62. | X |  |  |  |  |
| Wang C, La Salle T, Wu C, Do KA, Sullivan KE. School climate and parental involvement buffer the risk of peer victimization on suicidal thoughts and behaviors among Asian American middle school students. Asian American journal of psychology. 2018 Dec;9(4):296. | X | X | X |  |  |
| Gardella JH, Fisher BW, Teurbe-Tolon AR. A systematic review and meta-analysis of cyber-victimization and educational outcomes for adolescents. Review of Educational Research. 2017 Apr;87(2):283-308. | X |  |  |  |  |
| Holfeld B, Sukhawathanakul P. Associations between internet attachment, cyber victimization, and internalizing symptoms among adolescents. Cyberpsychology, Behavior, and Social Networking. 2017 Feb 1;20(2):91-6. | X |  |  |  |  |
| Lu Y, Van Ouytsel J, Walrave M, Ponnet K, Temple JR. Cross-sectional and temporal associations between cyber dating abuse victimization and mental health and substance use outcomes. Journal of Adolescence. 2018 Jun 1;65:1-5. | X |  |  |  |  |
| Rose CA, Tynes BM. Longitudinal associations between cybervictimization and mental health among US adolescents. Journal of Adolescent Health. 2015 Sep 1;57(3):305-12. | X |  |  |  |  |
| Steiner RJ, Rasberry CN. Brief report: Associations between in-person and electronic bullying victimization and missing school because of safety concerns among US high school students. Journal of adolescence. 2015 Aug 1;43:1-4. | X |  |  |  |  |
| Romero AJ, Bauman S, Borgstrom M, Kim SE. Examining suicidality, bullying, and gun carrying among Latina/o youth over 10 years. American journal of orthopsychiatry. 2018;88(4):450. | X |  |  |  |  |
| Wigderson S, Lynch M. Cyber-and traditional peer victimization: Unique relationships with adolescent well-being. Psychology of Violence. 2013 Oct;3(4):297. | X |  |  |  |  |
| Reed KP, Nugent W, Cooper RL. Testing a path model of relationships between gender, age, and bullying victimization and violent behavior, substance abuse, depression, suicidal ideation, and suicide attempts in adolescents. Children and youth services review. 2015 Aug 1;55:128-37. | X |  |  |  |  |
| Simckes MS, Simonetti JA, Moreno MA, Rivara FP, Oudekerk BA, Rowhani-Rahbar A. Access to a loaded gun without adult permission and school-based bullying. Journal of Adolescent Health. 2017 Sep 1;61(3):329-34. | X |  |  |  |  |
| Hase CN, Goldberg SB, Smith D, Stuck A, Campain J. Impacts of traditional bullying and cyberbullying on the mental health of middle school and high school students. Psychology in the Schools. 2015 Jul;52(6):607-17. | X |  |  |  |  |
| Evans CB, Smokowski PR, Cotter KL. Cumulative bullying victimization: An investigation of the dose–response relationship between victimization and the associated mental health outcomes, social supports, and school experiences of rural adolescents. Children and youth services review. 2014 Sep 1;44:256-64. | X |  |  |  |  |
| Goebert D, Else I, Matsu C, Chung-Do J, Chang JY. The impact of cyberbullying on substance use and mental health in a multiethnic sample. Maternal and child health journal. 2011 Nov;15(8):1282-6. | X |  |  |  |  |
| Hinduja S, Patchin JW. Bullying, cyberbullying, and suicide. Archives of suicide research. 2010 Jul 28;14(3):206-21. | X |  |  |  |  |
| Hinduja S, Patchin JW. Offline consequences of online victimization: School violence and delinquency. Journal of school violence. 2007 Oct 9;6(3):89-112. | X |  |  |  |  |
| Wang J, Nansel TR, Iannotti RJ. Cyber and traditional bullying: Differential association with depression. Journal of adolescent health. 2011 Apr 1;48(4):415-7. | X |  |  |  |  |
| Flannery DJ, Scholer SJ, Noriega I. Bullying and school violence. Pediatric Clinics. 2023 Dec 1;70(6):1153-70. | X |  | X |  |  |
| John A, Glendenning AC, Marchant A, Montgomery P, Stewart A, Wood S, Lloyd K, Hawton K. Self-harm, suicidal behaviours, and cyberbullying in children and young people: Systematic review. Journal of medical internet research. 2018 Apr 19;20(4):e9044. | X |  |  |  |  |
| Nagata JM, Yang JH, Singh G, Kiss O, Ganson KT, Testa A, Jackson DB, Baker FC. Cyberbullying and sleep disturbance among early adolescents in the US. Academic pediatrics. 2023 Aug 1;23(6):1220-5. | X |  |  |  |  |
| Christensen JB, Jones MS, Hoffmann JP. Bullying Victimization and Youth’s Likelihood of Carrying a Handgun. Journal of interpersonal violence. 2025 May;40(9-10):2031-54. | X |  |  |  |  |
| Wright MF, Wachs S. Adolescents’ psychological consequences and cyber victimization: The moderation of school-belongingness and ethnicity. International journal of environmental research and public health. 2019 Jul;16(14):2493. | X |  |  |  |  |
| Sumner SA, Ferguson B, Bason B, Dink J, Yard E, Hertz M, Hilkert B, Holland K, Mercado-Crespo M, Tang S, Jones CM. Association of online risk factors with subsequent youth suicide-related behaviors in the US. JAMA network open. 2021 Sep 20;4(9):e2125860. | X |  |  |  |  |
| Richard J, Marchica L, Ivoska W, Derevensky J. Bullying victimization and problem video gaming: The mediating role of externalizing and internalizing problems. International journal of environmental research and public health. 2021 Feb;18(4):1930. | X |  |  |  |  |
| Grinshteyn E, Tony Yang Y. The association between electronic bullying and school absenteeism among high school students in the United States. Journal of school health. 2017 Feb;87(2):142-9. | X |  |  |  |  |
| Duong J, Bradshaw C. Associations between bullying and engaging in aggressive and suicidal behaviors among sexual minority youth: The moderating role of connectedness. Journal of school health. 2014 Oct;84(10):636-45. | X |  |  |  |  |
| Pham TB, Adesman A. Increased risk of sadness and suicidality among victims of bullying experiencing additional threats to physical safety. International journal of adolescent medicine and health. 2020 Apr 28;32(2):20170109. | X |  |  |  |  |
| Predescu E, Calugar I, Sipos R. Cyberbullying and non-suicidal self-injury (NSSI) in adolescence: exploring moderators and mediators through a systematic review. Children. 2024 Mar 29;11(4):410. | X |  |  |  |  |
| Nguyen TH, Shah G, Muzamil M, Ikhile O, Ayangunna E, Kaur R. Association of in-school and electronic bullying with suicidality and feelings of hopelessness among adolescents in the United States. Children. 2023 Apr 21;10(4):755. | X |  |  |  |  |
| Elgar FJ, Napoletano A, Saul G, Dirks MA, Craig W, Poteat VP, Holt M, Koenig BW. Cyberbullying victimization and mental health in adolescents and the moderating role of family dinners. JAMA pediatrics. 2014 Nov;168(11):1015-22. | X | X |  |  |  |
| Garthe RC, Kim S, Welsh M, Wegmann K, Klingenberg J. Cyber-victimization and mental health concerns among middle school students before and during the COVID-19 pandemic. Journal of youth and adolescence. 2023 Apr;52(4):840-51. | X |  |  |  |  |
| Mantey DS, Yockey RA, Springer AE. Digital screen time and suicidality during high school: How important is cyberbullying? A mediation analysis using the youth risk behavioral surveillance survey, 2011–2019. Preventive medicine. 2023 Jan 1;166:107330. | X |  |  |  |  |
| McLoughlin LT, Simcock G, Schwenn P, Beaudequin D, Boyes A, Parker M, Lagopoulos J, Hermens DF. Social connectedness, cyberbullying, and well-being: preliminary findings from the longitudinal adolescent brain study. Cyberpsychology, Behavior, and Social Networking. 2022 May 1;25(5):301-9. | X |  |  |  |  |
| McField AA, Lawrence TI, Okoli IC. Examining the relationships between cyberbullying, relational victimization, and family support on depressive symptoms and substance use among adolescents. Clinical child psychology and psychiatry. 2023 Jan;28(1):224-36. | X | X |  |  |  |
| Srivastava A, Rusow J, Schrager SM, Stephenson R, Goldbach JT. Digital sexual violence and suicide risk in a national sample of sexual minority adolescents. Journal of interpersonal violence. 2023 Feb;38(3-4):4443-58. | X |  |  |  |  |
| Astatke M, Weng C, Chen S. A literature review of the effects of social networking sites on secondary school students’ academic achievement. Interactive Learning Environments. 2023 May 19;31(4):2153-69. | X |  |  |  |  |
| Dorol O, Mishara BL. Systematic review of risk and protective factors for suicidal and self-harm behaviors among children and adolescents involved with cyberbullying. Preventive medicine. 2021 Nov 1;152:106684. | X | X |  |  |  |
| Kwon M, Seo YS, Nickerson AB, Dickerson SS, Park E, Livingston JA. Sleep quality as a mediator of the relationship between cyber victimization and depression. Journal of nursing scholarship. 2020 Jul;52(4):416-25. | X |  |  |  |  |
| Chan SF, La Greca AM, Peugh JL. Cyber victimization, cyber aggression, and adolescent alcohol use: Short-term prospective and reciprocal associations. Journal of adolescence. 2019 Jul 1;74:13-23. | X |  |  |  |  |
| Humphries KD, Li L, Smith GA, Bridge JA, Zhu M. Suicide attempts in association with traditional and electronic bullying among heterosexual and sexual minority US high school students. Journal of Adolescent Health. 2021 Jun 1;68(6):1211-4. | X |  |  |  |  |
| Kreski NT, Chen Q, Olfson M, Cerdá M, Hasin D, Martins SS, Mauro PM, Keyes KM. Trends in adolescent online and offline victimization and suicide risk factors. Pediatrics. 2021 Sep 1;148(3):e2020049585. | X |  |  |  |  |
| Boccio CM, Leal WE, Jackson DB. Bullying victimization and nicotine and marijuana vaping among Florida adolescents. Drug and alcohol dependence. 2022 Aug 1;237:109536. | X |  |  |  |  |
| Doxbeck CR. Up in smoke: exploring the relationship between bullying victimization and e-cigarette use in sexual minority youths. Substance Use & Misuse. 2020 Oct 1;55(13):2221-9. | X |  |  |  |  |
| Díaz KI, Fite PJ. Cyber victimization and its association with substance use, anxiety, and depression symptoms among middle school youth. InChild & Youth Care Forum 2019 Aug 1 (Vol. 48, No. 4, pp. 529-544). New York: Springer US. | X |  |  |  |  |
| Baiden P, Tadeo SK. Investigating the association between bullying victimization and suicidal ideation among adolescents: Evidence from the 2017 Youth Risk Behavior Survey. Child abuse & neglect. 2020 Apr 1;102:104417. | X |  |  |  |  |
| Baiden P, Tadeo SK. Examining the association between bullying victimization and prescription drug misuse among adolescents in the United States. Journal of affective disorders. 2019 Dec 1;259:317-24. | X |  |  |  |  |
| Hinduja S, Patchin JW. Connecting adolescent suicide to the severity of bullying and cyberbullying. Journal of school violence. 2019 Jul 3;18(3):333-46. | X |  |  |  |  |
| DeMello AS, Peskin MF, Hill RM, Casarez RL, Santa Maria DM. The Impact of Bullying Victimization and Sexual Orientation on the Severity of Suicidal Behavior. Violence & Victims. 2022 Oct 1;37(5). | X |  |  |  |  |
| Priesman ER, Wright LE. Actions speak louder than words: examining the relationship between violent behaviors and bullying victimization among adolescents. Violence and gender. 2018 Dec 1;5(4):259-63. | X |  |  |  |  |
| DePaolis KJ, Williford A. Pathways from cyberbullying victimization to negative health outcomes among elementary school students: A longitudinal investigation. Journal of Child and Family Studies. 2019 Sep 1;28(9):2390-403. | X |  |  |  |  |
| Christina S, Magson NR, Kakar V, Rapee RM. The bidirectional relationships between peer victimization and internalizing problems in school-aged children: An updated systematic review and meta-analysis. Clinical psychology review. 2021 Apr 1;85:101979. | X |  |  |  |  |
| Espinoza G. The protective role of friends in the link between daily cyber victimization and adjustment problems among predominately Latino adolescents. Journal of Child & Adolescent Trauma. 2018 Mar;11(1):61-70. | X |  |  |  |  |
| Wright MF. Cyberstalking victimization, depression, and academic performance: The role of perceived social support from parents. Cyberpsychology, Behavior, and Social Networking. 2018 Feb 1;21(2):110-6. | X | X |  |  |  |
| Fredrick SS, Demaray MK. Peer victimization and suicidal ideation: The role of gender and depression in a school-based sample. Journal of school psychology. 2018 Apr 1;67:1-5. | X |  |  |  |  |
| Lee J, Choi MJ, Thornberg R, Hong JS. Exploring sex differences in the association between bullying involvement and alcohol and marijuana use among US adolescents in 6th to 10th grade. Substance use & misuse. 2020 May 1;55(8):1203-13. | X |  |  |  |  |
| Torres CE, D’Alessio SJ, Stolzenberg L. The effect of social, verbal, physical, and cyberbullying victimization on academic performance. Victims & offenders. 2020 Jan 2;15(1):1-21. | X |  |  |  |  |
| Brown CF, Demaray MK, Tennant JE, Jenkins LN. Cyber victimization in high school: Measurement, overlap with face-to-face victimization, and associations with social–emotional outcomes. School psychology review. 2017 Sep 1;46(3):288-303. | X |  |  |  |  |
| Lee J, Hong JS, Resko SM, Tripodi SJ. Face-to-face bullying, cyberbullying, and multiple forms of substance use among school-age adolescents in the USA. School mental health. 2018 Mar;10(1):12-25. | X |  |  |  |  |
| Wright MF. Cybervictimization and substance use among adolescents: The moderation of perceived social support. Journal of Social Work Practice in the Addictions. 2016 Apr 2;16(1-2):93-112. | X |  |  |  |  |
| Espinoza G. Daily cybervictimization among Latino adolescents: Links with emotional, physical and school adjustment. Journal of Applied Developmental Psychology. 2015 May 1;38:39-48. | X |  |  |  |  |
| Brown CF, Demaray MK, Secord SM. Cyber victimization in middle school and relations to social emotional outcomes. Computers in human behavior. 2014 Jun 1;35:12-21. | X |  |  |  |  |
| Yousef WS, Bellamy A. The impact of cyberbullying on the self-esteem and academic functioning of Arab American middle and high school students. Electronic Journal of Research in Educational Psychology. 2015;13(3):463-82. | X |  |  |  |  |
| Donoghue C, Meltzer LJ. Sleep it off: Bullying and sleep disturbances in adolescents. Journal of Adolescence. 2018 Oct 1;68:87-93. | X |  |  |  |  |
| Landoll RR, La Greca AM, Lai BS, Chan SF, Herge WM. Cyber victimization by peers: Prospective associations with adolescent social anxiety and depressive symptoms. Journal of adolescence. 2015 Jul 1;42:77-86. | X |  |  |  |  |
| Schneider SK, O'donnell L, Stueve A, Coulter RW. Cyberbullying, school bullying, and psychological distress: A regional census of high school students. American journal of public health. 2012 Jan;102(1):171-7. | X |  |  |  |  |
| Gini G, Espelage DL. Peer victimization, cyberbullying, and suicide risk in children and adolescents. Jama. 2014 Aug 6;312(5):545-6. | X |  |  |  |  |
| Hay C, Meldrum R. Bullying victimization and adolescent self-harm: Testing hypotheses from general strain theory. Journal of youth and adolescence. 2010 May;39(5):446-59. | X |  |  |  |  |
| Klomek AB, Sourander A, Gould M. The association of suicide and bullying in childhood to young adulthood: A review of cross-sectional and longitudinal research findings. The Canadian Journal of Psychiatry. 2010 May;55(5):282-8. | X |  |  |  |  |
| Armitage R. Bullying in children: impact on child health. BMJ paediatrics open. 2021 Mar 11;5(1):e000939. | X |  |  |  |  |
| Kreski NT, Chen Q, Olfson M, Cerdá M, Martins SS, Mauro PM, Branas CC, Rajan S, Keyes KM. Experiences of online bullying and offline violence‐related behaviors among a nationally representative sample of US adolescents, 2011 to 2019. Journal of school health. 2022 Apr;92(4):376-86. | X |  |  |  |  |
| Arnon S, Brunstein Klomek A, Visoki E, Moore TM, Argabright ST, DiDomenico GE, Benton TD, Barzilay R. Association of cyberbullying experiences and perpetration with suicidality in early adolescence. JAMA network open. 2022 Jun 27;5(6):e2218746. | X |  |  |  |  |
| Liu S, English D, Xiao Y, Li Y, Niu L. Sexual and gender minority identity, peer victimization, and suicidality in adolescents: A mediation study using the ABCD Study. Journal of Child Psychology and Psychiatry. 2025 Oct;66(10):1473-83. | X |  |  |  |  |
| Kuehn KS, Wagner A, Velloza J. Estimating the magnitude of the relation between bullying, e-bullying, and suicidal behaviors among United States youth, 2015. Crisis. 2018 Sep 14. | X |  |  |  |  |
| Messias E, Kindrick K, Castro J. School bullying, cyberbullying, or both: Correlates of teen suicidality in the 2011 CDC youth risk behavior survey. Comprehensive psychiatry. 2014 Jul 1;55(5):1063-8. | X |  |  |  |  |
| Holt M, Espelage D, Van Ryzin M, Bowman C. Peer victimization and sexual risk taking among adolescents. Journal of School Health. 2018 Dec;88(12):903-9. | X |  |  |  |  |
| Kim YK, Yu M, Cronley C, Yang M. Peer victimization, depression, and sexual risk behaviors among high school youth in the United States: a gender-based approach. International journal of adolescent medicine and health. 2022 Jun 1;34(3). | X |  |  |  |  |
| Kim YK, Kim YJ, Maleku A, Moon SS. Typologies of peer victimization, depression, and alcohol use among high school youth in the United States: Measuring gender differences. Social work in public health. 2019 May 19;34(4):293-306. | X |  |  |  |  |
| Nikolaou D. Does cyberbullying impact youth suicidal behaviors?. Journal of health economics. 2017 Dec 1;56:30-46. | X |  |  |  |  |
| Abreu RL, Kenny MC. Cyberbullying and LGBTQ youth: A systematic literature review and recommendations for prevention and intervention. Journal of child & adolescent trauma. 2018 Mar;11(1):81-97. | X |  |  |  |  |
| Doumas DM, Midgett A. Witnessing cyberbullying and suicidal ideation among middle school students. Psychology in the Schools. 2023 Apr;60(4):1149-63. | X |  |  |  |  |
| Holfeld B, Baitz R. The mediating and moderating effects of social support and school climate on the association between cyber victimization and internalizing symptoms. Journal of Youth and Adolescence. 2020 Nov;49(11):2214-28. | X | X |  |  |  |
